# Acute Q Fever in an Ankylosing Spondyloarthritis Patient Treated with Etanercept

**DOI:** 10.1155/2021/9944387

**Published:** 2021-06-01

**Authors:** A. Guiga, D. Khalifa, W. Ben Yahia, N. El Amri, A. Atig, N. Ghannouchi

**Affiliations:** ^1^Department of Internal Medicine, Farhat Hached Hospital, Sousse, Tunisia; ^2^Department of Rheumatology, Farhat Hached Hospital, Sousse, Tunisia

## Abstract

Q fever is a rare zoonotic infection caused by *Coxiella burnetii*. Tumor necrosis factor-alpha (TNF-*α*) has an important role in the early control of this infection. However, TNF-*α* blockers increase the risk of infectious diseases. We present herein a patient who developed acute Q fever under anti-TNF-*α* who had a good evolution after anti-TNF stoppage and treatment with doxycycline.

## 1. Introduction

Q fever is caused by the intracellular bacterium *Coxiella burnetii*. It is a rare infection affecting mainly farmers and people exposed to contaminated air or livestock. It can present as an acute infection with nonspecific symptoms or a chronic one [1]. Diagnosis is based on serologic tests while PCR assays are of better contribution [2]. TNF-*α* has an important role in the immune response against *C. burnetii* [3]. We present the case of a patient who was treated with anti-TNF-*α* for an ankylosing spondyloarthritis who developed acute Q fever.

## 2. Case Report

A 41-year-old male farmer with a history of chronic hepatitis B treated with entecavir, and ankylosing spondyloarthritis treated since last year with weekly etanercept injections, presented to our internal medicine ward for prolonged fever and malaise two weeks prior to evaluation. Upon investigation, he appeared to have chills and night sweats but denied having coughs, myalgias, bowel movement abnormalities, headaches, or any burning sensation with urination. On physical examination, he had a fever of 39°C, a 1 cm large, not painful, cervical lymph node, a stooped over posture, and a totally rigid spine. He had a blood pressure of 120/60 mmHg, and on cardiovascular examination, he had no murmur. Laboratory tests demonstrated a normal blood count, an elevated CRP of 244 mg/L, an accelerated erythrocyte rate of 37 mmH1, an inflammatory pattern on serum protein electrophoresis, and slightly elevated liver enzymes (AST = 46 UI/L and ALT = 36 UI/L) while liver and renal functions were normal. Blood cultures were negative and serologic tests for EBV, CMV, *Brucella*, HSV, HIV, and HCV were negative. Transthoracic cardiac ultrasound showed no signs of endocarditis. Antinuclear antibodies were positive (1:100), but anti-ENA antibodies were negative and without other signs of systemic lupus erythematosus. C3 and C4 complement fractions were normal.

Testing for hepatitis B surface antigen (HbsAg) and antibody to hepatitis B core antigen (anti-HBc) was positive and PCR test for HBV DNA showed a low viral load.

A computed tomography scan of the neck, chest, and abdomen showed a lymph node of the 4^th^ cervical chain but no abscesses or internal lymphadenopathy. By the end of the investigation, serologic diagnosis of Q fever appeared to be positive for both IgG and IgM. PCR for *C. burnetii* DNA was not performed. Treatment with etanercept was thus placed on hold and doxycycline 200 mg per day was prescribed for two weeks. The patient's physical condition improved as the fever stopped and he was checked on regularly for signs of progression to chronic Q fever. Liver enzymes were progressively back to normal as well as CRP levels that were negative during his last medical visit.

## 3. Discussion

Q fever is a rare zoonotic rickettsiosis caused by infection with *C. burnetii*. It spreads via aerosol transmission from contaminated soil or manipulation of infected livestock and its products. Other rare forms of transmission have been described such as oral consumption of raw milk, sexual transmission, and tick bites. It can present as either an acute or chronic disease. Individuals at risk of Q fever are males of age ≥ 40 years old, particularly farmers, veterinarians, slaughterhouse workers, and laboratory workers. Some conditions such as immunosuppressive therapy, preexisting valvulopathy, or vascular graft expose to a risk of chronic Q fever infection. Acute Q fever manifests as a febrile disease with nonspecific symptoms including headaches, myalgias, chills, and night sweats. However, up to 50% of patients with acute Q fever are asymptomatic. Other manifestations may include pneumonia and hepatitis, and less frequently, meningitis, encephalitis, pericarditis, myocarditis, and cholecystitis [1, 4].

Diagnosis of Q fever is based on the serologic test. IgM antibodies against phase II antigen are the marker of the acute infection while high levels against phase I antigen indicate a chronic infection. There is no consensus about serologic follow-up for acute infection, but serologic control after 6 or 9 months of the acute infection in people at high risk of chronic infection is highly recommended [2].

TNF-*α* has a major role in the defense against intracellular germs such as *C. burnetii*, as it has been proven that in mice; TNF-*α* suppression is responsible for severe heart lesions and early bacteremia [3, 5].

The literature regarding Q fever and biologics in spondyloarthritis is scarce. One case of acute Q fever in an ankylosing spondyloarthritis treated with infliximab has been reported as well as a case of skin psoriasis under etanercept manifesting with chronic Q fever [6]. As for rheumatoid arthritis (RA), two cases of chronic Q fever manifested as endocarditis have been reported in two women under etanercept and were known to have had previous cardiac valve abnormalities [7, 8].

A study during the outbreak of Q fever between 2007 and 2010 in the Netherlands, comparing rheumatoid arthritis (RA) patients treated with TNF-*α* blockers and anti-TNF-*α* naïve individuals, showed a similar prevalence among both groups for *C. burnetii* infection but with an increased frequency of chronic Q fever in anti-TNF-*α*-treated patients. These findings, although statistically insignificant, can be proof of the insufficient control of *C. burnetii* infection under anti-TNF-*α* blockers [9].

As for our patient, being treated with etanercept for a year could be an additional risk for developing Q fever other than his profession. Another mechanism could be IFN-*γ* depletion due to chronic hepatitis B infection. The role of IFN-*γ* in macrophage activation has been established against intracellular microorganisms such as *C. burnetii* as it promotes its killing in monocytes through an apoptotic mechanism mediated by TNF. Monocytic THP-1 cells infected with *C. burnetii* treated with IFN-*γ* had a reduced viability by 70% within 24 h of treatment with IFN-*γ* [10]. A Chinese study measuring IFN-*γ* expression in blood T cells with flow cytometry in chronic HBV showed that levels of IFN-*γ* expressed by T cells were reduced compared to healthy controls. However, during chronic HBV infection, treatment with entecavir was accompanied by the increase of IFN-*γ* level and IFN-*γ*/IL-4 ratio in CD8(+) T cells [11].

Treatment of Q fever is based on doxycycline and in combination with hydroxychloroquine in its chronic form. The duration of treatment depends on the presentation of the infection in its acute or chronic form [12]. Our patient was closely monitored for appearing signs of endocarditis and he was administered doxycycline 200 mg per day for 14 days. This case raises questions to the exact prevalence of Q fever infection among patients under TNF-*α* inhibitors and especially to those with ankylosing spondyloarthritis and comparison of Q fever infection risk among different TNF-*α*-blocking agents depending on their mechanism of action (monoclonal antibodies vs. circulating receptor fusion protein).

## Data Availability

The additional data are available from the corresponding author upon request.
